# Complete linear mitochondrial genomes for *Cephea cephea* and *Mastigias albipunctata* (Scyphozoa: Rhizostomeae), with an analysis of phylogenetic relationships

**DOI:** 10.1080/23802359.2024.2429644

**Published:** 2024-11-14

**Authors:** Kei Chloe Tan, Cheryl Lewis Ames, Allen G. Collins

**Affiliations:** a Graduate School of Agricultural Sciences & WPI-Advanced Institute for Marine Ecosystem Change, Tohoku University, Sendai, Japan; b National Systematics Laboratory; Office of Science and Technology, NOAA National Marine Fisheries Service, Washington, DC, USA; c Department of Invertebrate Zoology, Smithsonian National Museum of Natural History (NMNH), Washington, DC, USA

**Keywords:** Jellyfishes, Kolpophorae, Krykomyaria, linear mitochondrial genomes, phylogeny

## Abstract

Jellyfishes of the order Rhizostomeae include ecologically and economically important species predisposed to forming massive aggregations. This study reports the first complete mitochondrial genome of two rhizostomes: *Cephea cephea* (Forskål, 1775) and *Mastigias albipunctata* Stiasny, 1920. The linear mitochondrial genomes are 16,667bp and 16,707bp in length and 65.5% and 68.4% AT respectively; each comprises 15 protein-coding genes (PCGs; dpo, orf314, cox1-3, nd1-6, nd4L, atp6, atp8 and cytB), two ribosomal RNAs (16S and 12S rRNA), and two tRNAs (trnM and trnW). The phylogenetic analysis reveals strong support for monophyly of the suborder Kolpophorae and *Cephea cephea* of the infraorder Actinomyaria as sister to Krykomyaria which includes *Mastigias*.

## Introduction

Jellyfishes of the order Rhizostomeae (class: Scyphozoa) have variable medusa morphologies, including the benthic ‘upside-down jellyfish’ *Cassiopea*, a model system (Ohdera et al. 2018), the pelagic *Mastigias*, and blooming species exploited for commercial fisheries and aquaculture (Omori and Nakano 2001; Brotz et al. 2017).

Rhizostomeae medusae lack tentacles on the umbrella margin but bear eight oral arms extending from the central region of the subumbrella. Some species have endosymbiotic relationships with Symbiodiniaceae, a family of dinoflagellates (Djeghri et al. 2019). Species of five genera, including *Cassiopea* and *Netrostoma*, a close relative of *Cephea*, are known to release motile, multicellular bodies comprising ciliated and stinging cells, termed ‘cassiosomes’, into the water column (Ames et al. 2020).

**Figure 1.**
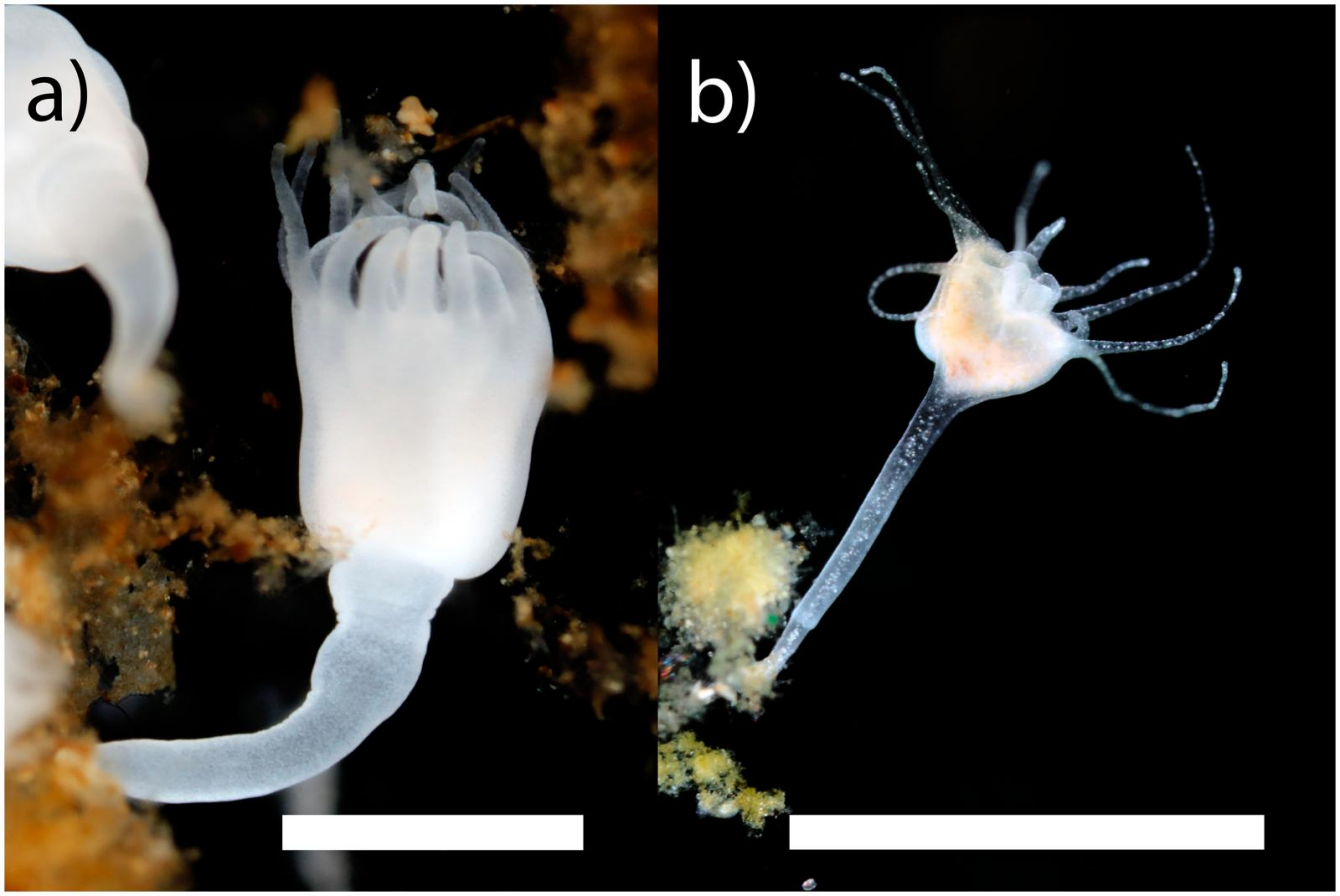
Photographs of the polyps of a) *cephea cephea* and b) *Mastigias albipunctata* reared in the AquaRoom, taken by Allen Collins. Scalebars represent 500 uM.

Rhizostomeae has 2 suborders, Dactyliophorae and Kolpophorae, with species in the latter often hosting Symbiodiniaceae. Apart from Kolpophorae genera *Cassiopea* and *Mastigias*, Dactyliophorae taxa have been sampled comparatively more thoroughly for molecular analysis. Accordingly, no genomic data are available for the Kolpophorae *Cephea cephea* or kin, precluding our understanding of the complete mitochondrial genome – a dynamic molecular marker for metazoan evolutionary studies. Hence, we sequenced the complete mitogenome of *Cephea cephea* (Forskål, 1775) and *Mastigias albipunctata* Stiasny, 1920 (Figure 1) and conducted phylogenetic analysis of the order Rhizostomeae.

## Materials and methods

Clonal polyp cultures of *Cephea cephea* and *Mastigias albipunctata* are kept live in the National Museum of Natural History (NMNH) Department of Invertebrate Zoology AquaRoom. Polyp specimens were deposited at the NMNH (https://naturalhistory.si.edu/, contact person: Allen Collins, e-mail: Allen.Collins@noaa.gov) under the vouchers USNM 1715474 and USNM 1715470 respectively. DNA from several polyps of each species was extracted using an AutoGenPrep 965 robot (AutoGen, Holliston, MA, USA) following the manufacturer’s tissue protocol. For whole genome shotgun sequencing, enzymatically sheared libraries were prepared with NEB Ultra II FS DNA library prep kit (New England Biolabs), targeting an insert size of approximately 400 bp. Libraries were amplified using six cycles of PCR following the kit manufacturer’s chemistry and thermocycler recommendations. We employed iTru y-yoke adapter stubs and iTru unique dual indices (Glenn et al. 2019) rather than NEB adapters and indices, tailoring the amount of adapter based on DNA concentrations specified in the NEB guidelines. Sequencing of equimolar pooled libraries (150 bp, paired end reads) was achieved using a NovaSeq 6000 (Illumina Inc., San Diego, CA, USA).

The resulting reads were trimmed of adapters and poor-quality sequences using BBDUK, part of BBTools (Bushnell B. – sourceforge.net/projects/bbmap/). To assemble mitochondrial genomes, we used the ‘Map to Reference’ function and built-in mapper of Geneious Prime 2022 (https://www.geneious.com), with sensitivity set to ‘medium/low’ and iterations set to 3 or 5, starting with GenBank published sequences for mitochondrial genes 16S rRNA (KY610618) and COX 1 (KU900928) for *Cephea cephea* and *Mastigias albipunctata*, respectively. Results of the pending mitochondrial genome assemblies were inspected and ends trimmed (up to 50 bp) where coverage was low (<5X). Consensus sequences were generated and used as subsequent reference seeds, and the ‘Map to Reference’ step was repeated until assemblies ceased to increase in size. Refer to Supplementary Figure 1 for read coverage plot.

Mitogenome annotation was performed in Geneious Prime 2024 (https://www.geneious.com), using genetic code 4, ‘Mold/Protozoan Mitochondrial’. Predicted open reading frames were checked manually by aligning to whole mitochondrial genomes of Rhizostomeae available in GenBank and annotated accordingly. tRNAs were identified using tRNAscan-SE v2.0 (http://lowelab.ucsc.edu/tRNAscan-SE/ (accessed 24 April 2024) with the default settings (Mold & Protozoa Mito genetic code) (Lowe and Chan 2016). rRNA genes were identified based on alignments with the most closely related species (*Mastigias papua* and *Phyllorhiza punctata*. Likewise, we annotated the UNVERIFIED mitogenomes of *Pseudorhiza haeckeli* (OZ032132)*, Catostylus mosaicus* (OZ025205) and a *Mastigias papua* (OZ025288) from GenBank.

Phylogenetic analysis employed our two newly generated whole mitochondrial genomes, and 12 complete and four partial mitochondrial genomes from GenBank (Table S1). In the absence of genomes for *Versuriga anadyomene, Thysanostoma thysanura* and *Lobonema smithii* their mitochondrial genes 16S rRNA and COX 1 were substituted to ensure representation of all known Rhizostomeae families (validated in WoRMS, https://www.marinespecies.org); the outgroup was Semaeostome *Aurelia aurita* (DQ787873). A maximum likelihood phylogenetic tree (TIM2 + F + G + I) was constructed in IQ-TREE (Trifinopoulos et al. 2016) using concatenated alignments (MAFFT alignment; Katoh and Standley 2013) of each protein-coding and rRNA gene sequence. Nodes were assessed using aBayes test and 1000 ultrafast bootstrap replicates (Hoang et al. 2018).

## Results

**Figure 2.**
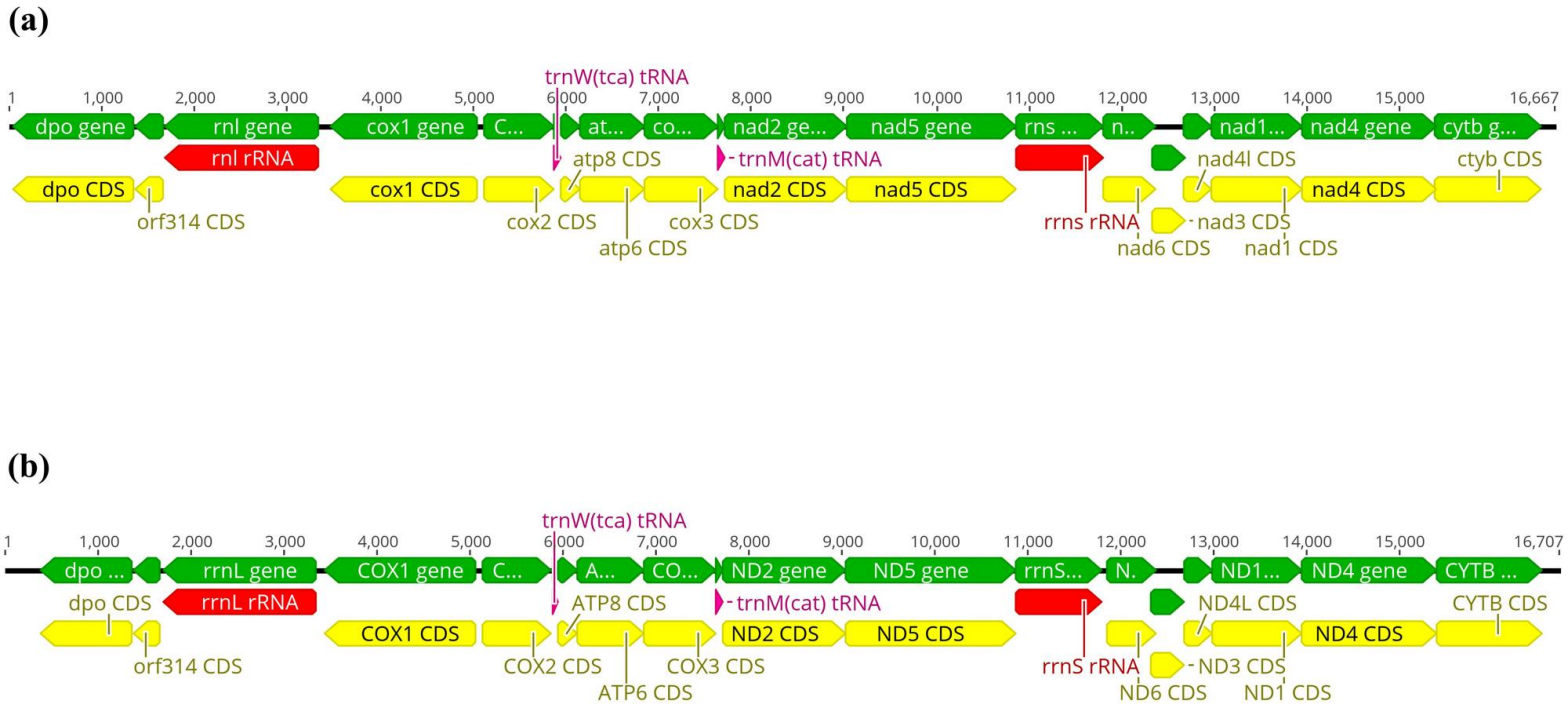
Graphic view of the linear mitochondrial genomes of a) *Cephea cephea* and b) *Mastigias albipunctata*. Annotated genes (n = 19) with their transcription direction indicated by arrowhead. Arrow colors: green = genes, yellow = coding regions (CDS), red = rRNA genes, pink = tRNA genes.

The complete mitogenomes of *Cephea cephea* and *Mastigias albipunctata* were 16,667 and 16,707bp in length, respectively, containing 15 protein-coding genes, two transfer RNA (tRNA) genes (trnW and trnM), and two ribosomal RNA (rRNA) genes (Figure 2a,b). Accessioned SRA datasets (BioSamples: SAMN41149076-7) for *C. cephea* and *M. albipunctata* contain 9,998,268 and 10,870,791 spots, respectively, with 101,434 and 182,567 reads mapping to the respective mitochondrial genomes with 860X and 1568X coverage.

For *C. cephea,* start codons for the protein-coding genes comprise 3 TAC, 11 ATG and 1 GTG; stop codons are 2 ATT, 3 TAG and 10 TAA (Figure 2a), whereas for *M. albipunctata,* start codons include 3 TAC, 10 ATG and 2 GTG; stop codons comprised 1 ATT, 1 ATC, 4 TAG and 9 TAA (Figure 2b). Base compositions were A: 29.2%, C: 16.5%, G: 18.0%, T: 36.3% for *C. cephea* and A: 30%, C: 14.8%, G: 16.8%, T: 38.4% for *M. albipunctata.* 12S rRNA and 16S rRNA were predicted to be 934 bp and 1665 bp, and 935 bp and 1645 bp for *C. cephea* and *M. albipunctata,* respectively.

**Figure 3.**
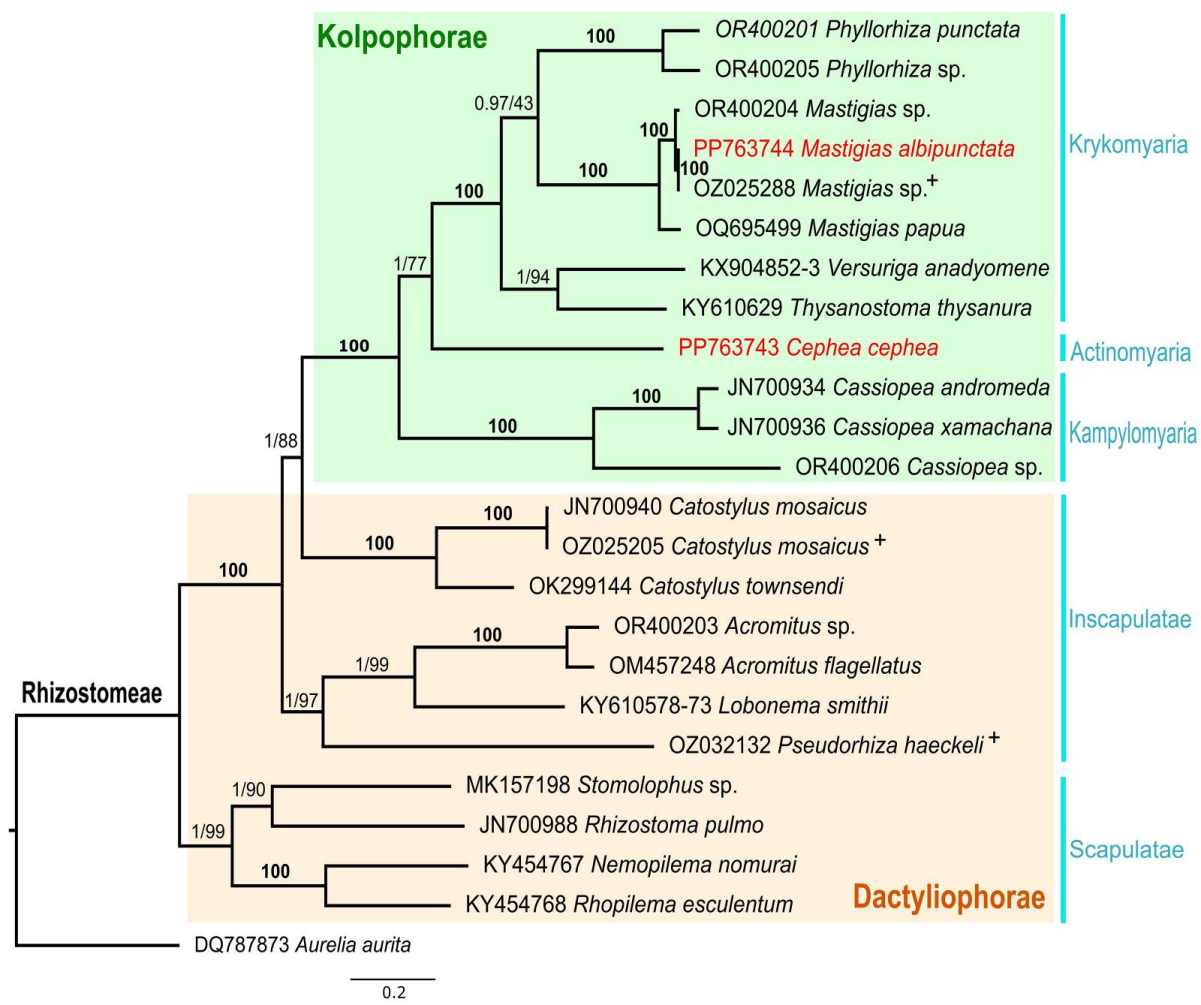
Maximum likelihood phylogenetic tree with aBayes and bootstrap support for the mitochondrial genomes of 24 rhizostomes and one semaeostomeae (*Aurelia aurita*) outgroup. Whole or partial mitochondrial genomes were not available for three families: Versurigidae, lobonematidae and thysanostomatidae. In this phylogenetic analysis the concatenated sequences of mitochondrial genes 16S rRNA and COX1 for *Versuriga anadyomene* (KX904852, KX904853) and *Lobonema smithii* (KY610578, KY610573) were used, while only 16S rRNA was used for *Thysanostoma thysanura* (KY610629). *Cephea cephea* (PP763743) and *Mastigias albipunctata* (PP763744) are highlighted in red and + denotes additional mtGenomes annotated in this study. aBayes support and ultrafast bootstrap support (%) (1000 replicates) were calculated for the nodes. Bold **100** represents indices of 100 for both criteria.

The resulting maximum likelihood tree (Figure 3) consisted of 24 rhizostome taxa with representatives from each family. Tree topology (maximal aBayes and bootstrap support) was consistent with the hypotheses of Uchida (1926) and Bayha et al. (2010) of monophyly of the suborder Kolpophorae derived from within Dactyliophorae. Monophyly of the infraorders Kampylomyaria, Krykomyaria, and Scapulatae were strongly supported (>95% bootstrap). *C. cephea* was placed as sister to the infraorder Krykomyaria.

## Discussions and conclusions

These two new mitochondrial genomes bring the number of accessioned annotated whole Rhizostomeae mitochondrial genomes to 14. Our mitogenome for *Mastigias albipunctata* fell into a distinct clade with so-called ‘*M. papua*’ (OZ025288) with 99.041% similarity, but only 90.861% identity to *M. papua* (OQ695499). A recent integrative analysis of *Mastigias* identified three phylogeographically and morphologically distinct lineages: *M. papua*, *M. albipunctata*, and *Mastigias* from Tufi (Souza and Dawson 2018). To assess the identities of our *Mastigias* exemplars, we reconstructed a COX 1 ML-phylogenetic tree (Supplementary Figure 2; Accession numbers in Table S2) in IQ-TREE with additional data from NCBI GenBank. Results corroborate the existence of three distinct clades, with NMNH live cultures in the *M. albipunctata* clade. Though OZ025288 is accessioned in GenBank under the species name *M. papua,* our findings support its proper identity as *M. albipunctata*.

Our analyses (Supplementary Figure 3) uncovered other likely errors in taxon identity. For instance, Ling et al. (2023) noted uncertainty in the identity of the juveniles from which they derived the sequences accessioned as *Phyllorhiza punctata* (OR400204) and *Acromitus* sp. (OR400205), which we discovered correspond to *Mastigias* and *Phyllorhiza* respectively.

Phylogenetic analysis corroborates previous studies that strongly support the monophyly of Kolpophorae, derived from within Dactyliophorae, highlighting the need for broader genomic sequencing of Rhizostomeae genera to fill data gaps. A similar phylogenetic approach conducted on the endosymbiotic Symbiodiniaceae DNA extracted from the rhizostome host offers a tractable method to explore patterns of Cnidaria-Dinoflagellate coevolution.

## Supplementary Material

Supplementary_Materials.docx

## Data Availability

The genome sequence data supporting this study’s findings are available at GenBank (https://www.ncbi.nlm.nih.gov/) under accession number PP763743-PP763744. The associated BioProject, SRA and Bio-Sample numbers are PRJNA1077907, SRR28865914-5 and SAMN41149076-7 respectively. Supplementary materials (https://doi.org/10.5281/zenodo.13961219) and our annotations of three other rhizostome mitochondrial genomes in GenBank are available here: https://doi.org/10.5281/zenodo.11580939.
