# Supplementary material for: Complete linear mitochondrial genomes for Cephea cephea and Mastigias albipunctata (Scyphozoa: Rhizostomeae), with an analysis of phylogenetic relationships
Source: Mitochondrial DNA B Resour. 2024 Nov 14;9(11):1544–8. doi: 10.1080/23802359.2024.2429644 (PMC11565657; doi:10.1080/23802359.2024.2429644)
Supplement: Supplementary_Materials.docx [file TMDN_A_2429644_SM7703.docx]

**Figure S1. Read coverage plot of a) *Cephea cephea* and b) *Mastigias albipunctata* mitogenomes.**

**Table S1. GenBank accessions of all Rhizostomeae mitochondrial genomes available in NCBI GenBank (accessed April 2024) and used to construct phylogenetic tree (Figure 3)**

| **GenBank Accession** | **Species** | **Locality** | **Literature** |
| --- | --- | --- | --- |
| (JN700934) | *Cassiopea andromeda* | Tiahura in Moorea (French Polynesia) | Kayal et al. 2012 |
| JN700936 | *Cassiopea xamachana* | unknown | Kayal et al. 2012 |
| (JN700940) | *Catostylus mosaicus* | unknown | Kayal et al. 2012 |
| (JN700988) | *Rhizostoma pulmo* | unknown | Kayal et al. 2012 |
| KY454767 | *Nemopilema nomurai* | Jiaozhou Bay, China | Wang and Sun 2017 |
| KY454768 | *Rhopilema esculentum* | Jiaozhou Bay, China | unpublished |
| MK157198 | *Stomolophus* sp. | unknown | unpublished |
| OK299144 | *Catostylus townsendi* | Yangxi, Donghai Sea, China | unpublished |
| OM457248 | *Acromitus flagellatus* | China | Lin et al. 2022 |
| OQ695499 | *Mastigias papua* | China | unpublished |
| OR400201 | *Phyllorhiza punctata* | Singapore | Ling et al. 2023 |
| (OR400203) | *Acromitus* sp. | Singapore | Ling et al. 2023 |
| OR400204 | *Mastigias* sp.*** | Singapore | Ling et al. 2023 |
| OR400205 | *Phyllorhiza* sp. *^Δ^* | Singapore | Ling et al. 2023 |
| OR400206 | *Cassiopea* sp. | Singapore | Ling et al. 2023 |
| OZ032132 | *Pseudorhiza haeckeli* ^+^ | unknown | unpublished |
| OZ025205 | *Catostylus mosaicus* ^+^ | unknown | unpublished |
| OZ025288 | *Mastigias* sp.* ^+^ | Japan | unpublished |
| PP763744 | *Mastigias albipunctata* ^+^ | unknown | This study |
| PP763743 | *Cephea cephea* ^+^ | unknown | This study |

*Note.* Accession numbers in brackets are partial genomes and were not used for annotation comparison.

* Samples renamed to *Mastigias* sp. from *Mastigias papua* and *Phyllorhiza punctata* respectively.

*^Δ^* Samples renamed to *Phyllorhiza* sp. from *Acromitus* sp.

+ MtGenomes annotated in this study.

**
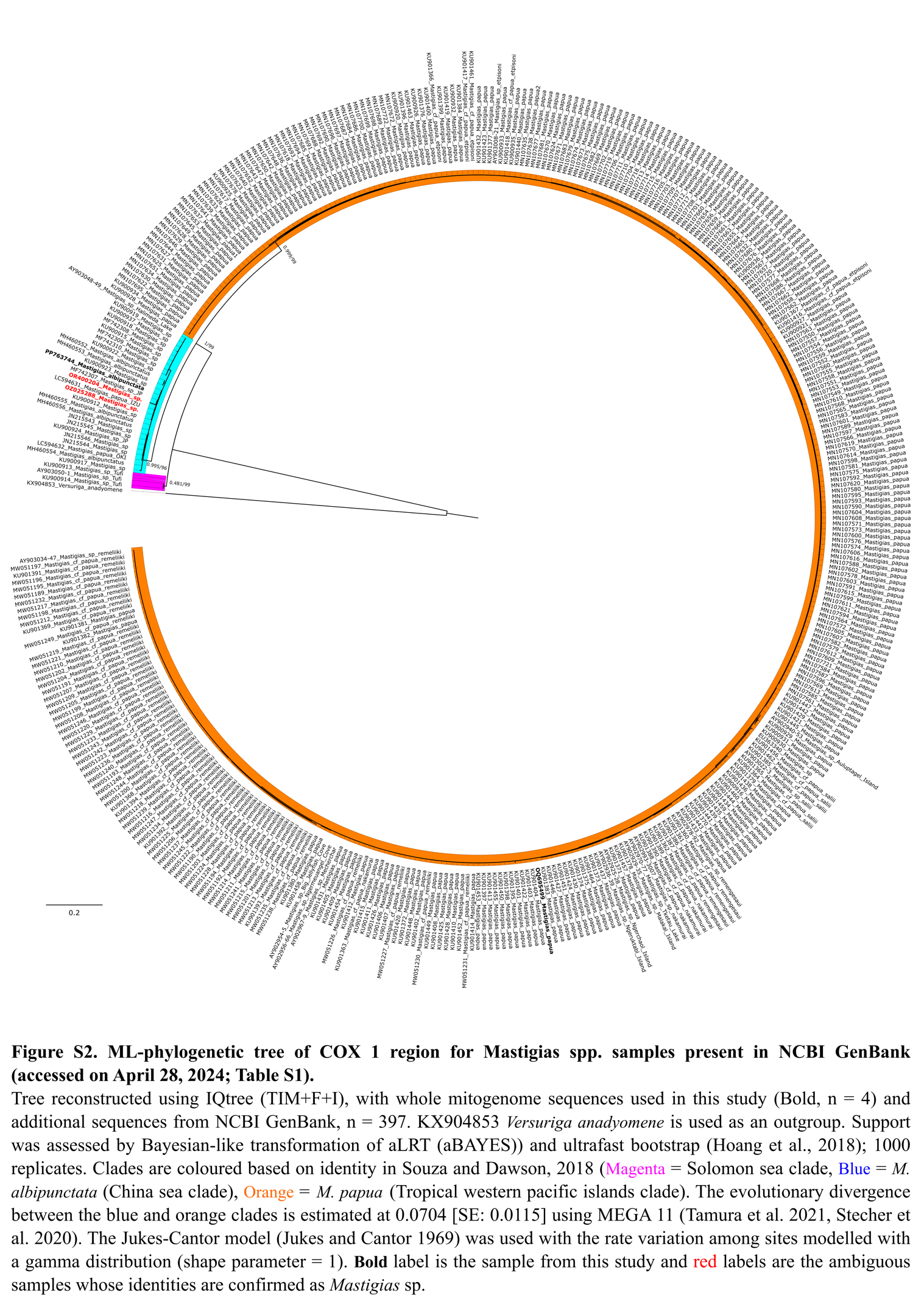
**

**Figure S2. ML-phylogenetic tree of COX 1 region for *Mastigias* samples present in NCBI GenBank (accessed on April 28, 2024; Table S1).**

Tree reconstructed using IQtree (TIM+F+I), with whole mitogenome sequences used in this study (Bold, n = 4) and additional sequences from NCBI GenBank, n = 397. KX904853 *Versuriga anadyomene* is used as an outgroup. Support was assessed by Bayesian-like transformation of aLRT (aBAYES)) and ultrafast bootstrap; 1000 replicates. Clades are coloured based on identity in Souza and Dawson, 2018 (Magenta = Solomon Sea clade, Blue = *M. albipunctata* (China sea clade), Orange = *M. papua* (Tropical western pacific islands clade). The evolutionary divergence between the blue and orange clades is estimated at 0.0704 [SE: 0.0115] using MEGA 11 (Tamura et al. 2021, Stecher et al. 2020). The Jukes-Cantor model (Jukes and Cantor 1969) was used with the rate variation among sites modelled with a gamma distribution (shape parameter = 1). **Bold** labels are samples used in this study and red labels are the ambiguous samples whose identities are confirmed as *Mastigias* sp.


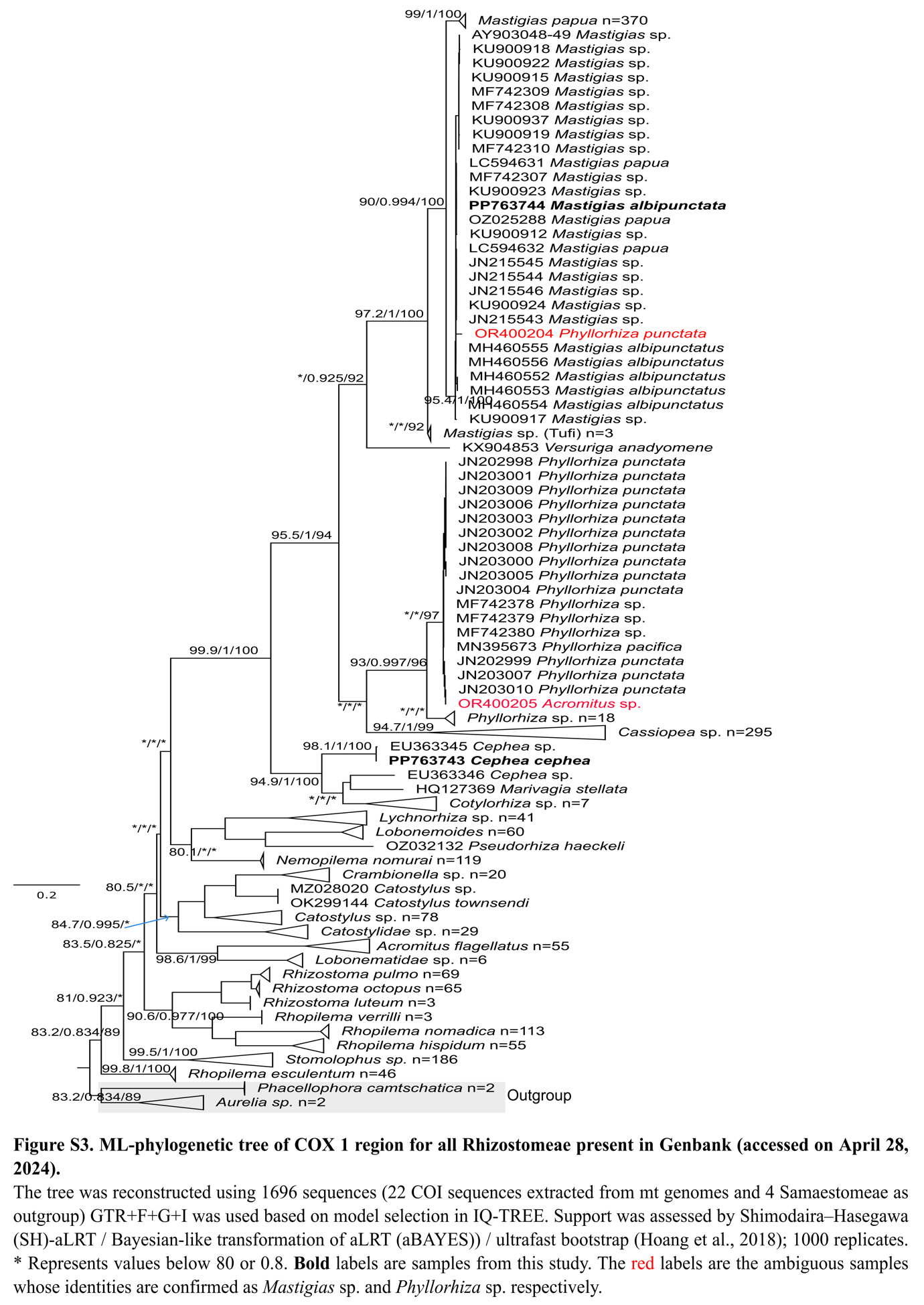


**Figure S3. ML-phylogenetic tree of COX 1 region for all Rhizostomeae present in Genbank (accessed on April 28, 2024).**

The tree was reconstructed using 1696 sequences (22 COI sequences extracted from mt genomes and 4 Semaeostomeae as outgroup) GTR+F+G+I was used based on model selection in IQ-TREE. Support was assessed by Shimodaira–Hasegawa (SH)-aLRT / Bayesian-like transformation of aLRT (aBAYES)) / ultrafast bootstrap;1000 replicates. * Represents values below 80 or 0.8. **Bold** labels are samples from this study. The red labels are the ambiguous samples whose identities are confirmed as Mastigias sp. and Phyllorhiza sp. respectively.
